# 

**DOI:** 10.1055/s-0035-1570106

**Published:** 2016-01

**Authors:** Agnaldo Lopes da Silva Filho, Jesus Paula Carvalho

**Affiliations:** 1 Departamento de Ginecologia e Obstetrícia da Universidade Federal de Minas Gerais, Belo Horizonte, MG, Brasil; 2 Disciplina de Ginecologia da Faculdade de Medicina da Universidade de São Paulo, São Paulo, SP, Brasil; 3 Serviço de Ginecologia Oncológica do Instituto do Câncer do Estado de São Paulo, São Paulo, SP, Brasil

## Importância do Câncer Ginecológico no Brasil

O câncer ginecológico tem uma alta incidência e é causa relevante de morbidade e óbitos no Brasil e no mundo. O Instituto Nacional do Câncer (INCA) estima a ocorrência de aproximadamente 30.000 novos casos por ano de câncer de colo uterino, corpo e ovário no Brasil. 1 Esses tumores são responsáveis por pelo menos 10% de todas as neoplasias malignas nas mulheres brasileiras, exceto o câncer de pele não melanoma ( Tabela 1 ). A abordagem do câncer ginecológico é complexa e ampla compreendendo ações preventivas, diagnósticas e terapêuticas que podem ser realizadas por médicos generalistas, ginecologistas e obstetras e outros especialistas. Porém existem ações e procedimentos de alta complexidade que demandam conhecimentos e treinamentos específicos, que só podem ser assimilados através de programas de treinamento em situações e em Instituições que dispõem de recursos para fornecer estes treinamentos. Dessa forma, é fundamental que ginecologistas e obstetras possam se qualificar para prestar assistência em Ginecologia Oncológica de Alta Complexidade.

**Tabela 1:** Incidência dos cânceres ginecológicos no Brasil – Estimativa 2014

Localização 1 ^a^	Casos Novos	%
Colo do útero	15.590	5,7
Corpo do útero	5.900	2,2
Ovário	5.680	2,1
Total	27.170	10
Fonte: INCA 1		

## Histórico e Definições da Ginecologia Oncológica no Mundo

A *European Society of Gynecologic Oncology* (ESGO) define o Ginecologista Oncológico como um especialista em Ginecologia e Obstetrícia com treinamento e capacidade para: avaliar e abordar de forma abrangente pacientes com câncer ginecológico ou de mama. Na União Europeia, o ginecologista habitualmente conduz o tratamento para câncer de mama, com exceção da Dinamarca, Finlândia, Irlanda, Holanda e Reino Unido; conduzir tratamento clínico e/ou cirúrgico das neoplasias malignas do trato genital feminino e mama (procedimentos cirúrgicos complexos abdominais) e praticar Ginecologia Oncológica em uma instituição em que estejam disponíveis todas as modalidades para tratamento oncológico, incluindo ainda procedimentos diagnósticos, terapêuticos e seguimento das pacientes.

A Ginecologia Oncológica é uma especialidade reconhecida nos Estados Unidos desde 1969 o que resultou em uma melhora significativa dos resultados nas mulheres com câncer ginecológico. 2 Esse profissional deve conduzir tratamento clínico e/ou cirúrgico das neoplasias malignas do trato genital feminino e praticar Ginecologia Oncológica em um contexto multidisciplinar. A sua formação deve ser direcionada para o câncer ginecológico, com conhecimentos específicos sobre a fisiopatologia, biologia tumoral, patologia, radioterapia, quimioterapia e cuidados paliativos. Esse profissional necessita um treinamento direcionado para aquisição de habilidades cirúrgicas avançadas.

A duração da formação em Ginecologia Oncológica é de três anos nos Estados Unidos e Reino Unido e de quatro anos na Alemanha. Segundo a ESGO, a duração da formação de subespecialidade deve incluir um mínimo de dois anos em um programa aprovado e deve incluir atividades clínicas e de pesquisa nas seguintes áreas: treinamento cirúrgico em unidade de oncologia, treinamento de Cirurgia Geral, Urologia, Radioterapia, Oncologia Clínica, Patologia e Citopatologia, Psico-oncologia e Biologia Tumoral. Exige ainda um número mínimo de cirurgias para obtenção do título na subespecialidade: 30 casos de cirurgia para câncer de endométrio, ovário ou tuba uterina; quinze casos de histerectomia radical; cinco casos de cirurgias para outras malignidades e pelo menos cinco casos de vulvectomia com linfadenectomia.

## Ginecologia Oncológica no Programa de Residência Médica de Ginecologia e Obstetrícia no Brasil

O treinamento em Ginecologia Oncológica é exigente, demanda tempo, recursos e dedicação em centros especializados. Novas tecnologias são incorporadas constantemente e novos paradigmas são incorporados aos protocolos de maneira cada vez mais rápida. O tempo de treinamento é muito maior do que as poucas semanas de treinamento específicos inseridas nos programas de residência médica em Obstetrícia e Ginecologia, que se mostram insuficientes para que os egressos possam prestar uma assistência adequada às mulheres com câncer ginecológico.

A inexistência de uma área de atuação em Ginecologia Oncológica de Alta Complexidade impossibilita a criação de programas de residência específicos nesta área com um a dois anos de duração. Com exceção da Ginecologia e Obstetrícia, todas as áreas básicas, incluindo a Clínica Médica, Cirurgia Geral e Pediatria, estão representadas na Cancerologia com a suas respectivas subespecialidades. A residência médica é a melhor forma de treinamento em especialidades, principalmente em áreas com vários níveis de complexidade e combinação de conhecimentos clínicos e cirúrgicos. Este é precisamente o caso da Ginecologia Oncológica de Alta Complexidade. A residência médica pode propiciar uma uniformidade na formação dos profissionais e consequentemente melhores resultados para as pacientes.

## Ginecologia Oncológica como Área de Atuação

A Ginecologia Oncológica é uma especialidade reconhecida em praticamente todos os países que apresentam uma assistência médica de qualidade, com exceção do Brasil, México e Rússia. De forma geral as três áreas de atuação mais reconhecidas são a Ginecologia Oncológica, seguida pela Medicina Fetal, Reprodução Humana e Uroginecologia. A Tabela 2 mostra as subespecialidades reconhecidas nos EUA, Alemanha e Reino Unido, países com um padrão de Medicina de alto nível. No Brasil a situação é bem divergente, a Associação Médica Brasileira (AMB) reconhece como áreas de atuação em Ginecologia e Obstetrícia a Densitometria Óssea, Endoscopia Ginecológica, Mamografia, Medicina Fetal, Reprodução Humana, Sexologia e Ultrassonografia em Ginecologia e Obstetrícia. O objetivo de se criar áreas de atuação consiste na necessidade de formação complementar em áreas complexas cuja curva de aprendizado não se conclui durante a residência médica em áreas básicas. As áreas de atuação quase sempre podem ser exercidas por mais de uma especialidade. No caso da Ginecologia Oncológica, considera-se a cancerologia cirúrgica uma especialidade afim para essa área de atuação.

**Tabela 2:** Áreas de atuação reconhecidas nos EUA, Alemanha, Reino Unido e Brasil

Áreas de atuação	EUA	Alemanha	Reino Unido	Brasil
Ginecologia oncológica (GO)	✓	✓	✓	
Medicina materno-fetal	✓	✓	✓	✓
Medicina reprodutiva	✓	✓	✓	✓
Uroginecologia	✓	✓	✓	
Densitometria óssea				✓
Endoscopia Ginecológica				✓
Mamografia				✓
Sexologia				✓
Ultrassonografia em GO				✓

A Ginecologia Oncológica tem vários níveis de complexidade durante o processo de diagnóstico e tratamento. 3 4 Houve uma melhora significativa dos resultados, incluindo sobrevida, nas pacientes com câncer de colo uterino, endométrio, vulva e principalmente câncer de ovário, tratadas por um ginecologista oncológico. 3 5 6 7 8 Tem ocorrido um movimento na última década pela centralização do tratamento do câncer em centros especializados. Isto vem a partir do reconhecimento de que o atendimento multidisciplinar, incluindo o acesso às avaliações do ginecologista oncológico, radioterapêuta e oncologista clínico, pode melhorar os resultados dos pacientes. 3 4 9 10

## Considerações Finais

Ressaltamos a necessidade de garantir que o médico ginecologista e obstetra, mantenha a sua atuação no rastreamento das neoplasias e condução do tratamento dos casos de menor complexidade. A Tabela 3 mostra um sumário das razões para criação da área de atuação em Ginecologia Oncológica. Após reconhecimento da área de atuação em Ginecologia serão definidos os critérios para certificação, assim como a adoção de regras transitórias para os profissionais que já atuam nessa área. A criação da área de atuação vai possibilitar oferecer anos adicionais de residência médica em Ginecologia e Obstetrícia com atuação em Ginecologia Oncológica de Alta Complexidade. Longe de ser uma atitude excludente, a Ginecologia Oncológica de Alta Complexidade pretende promover os mecanismos para que ginecologista e obstetras de todo o pais adquiram qualificação adequada para atuar em toda a cadeia de eventos que envolve a prevenção, o diagnóstico e o tratamento do câncer ginecológico, inclusive os procedimentos de alta complexidade.

**Tabela 3:** Razões para criação da área de atuação em Ginecologia Oncológica

**1**	O câncer ginecológico é muito prevalente: atinge aproximadamente 30.000 brasileiras a cada ano (10% de todas as neoplasias malignas no sexo feminino);
**2**	A abordagem do câncer ginecológico é complexa e requer um profissional capacitado, com conhecimentos específicos sobre a fisiopatologia, biologia tumoral, patologia, radioterapia e quimioterapia; além de habilidades cirúrgicas avançadas;
**3**	A Ginecologia Oncológica consiste em uma área de atuação extremamente consolidada há mais de 45 anos, sendo reconhecida em praticamente todos os grandes países. As áreas de atuação em Ginecologia e Obstetrícia reconhecidas pela Associação Médica Brasileira (AMB) são absolutamente discrepantes com os outros países e não inclui a Ginecologia Oncológica;
**4**	O treinamento durante a residência médica em Obstetrícia e Ginecologia é insuficiente para prestar uma assistência adequada às mulheres com câncer ginecológico. O não reconhecimento da Ginecologia Oncológica representa uma enorme barreira para a formação complementar desses profissionais;
**5**	Mulheres com câncer ginecológico assistidas por um Ginecologista Oncológico apresentam melhor prognóstico.
